# Sternal Tumor Resection and Complex Chest Wall Reconstruction of a Solitary Plasmacytoma: A Report of a Rare Case

**DOI:** 10.7759/cureus.89342

**Published:** 2025-08-04

**Authors:** Magdalena Alexieva, Georgi Gergov, Evgeni V Mekov, Silvia Ivanova, Rosen E Petkov, Georgi Yankov

**Affiliations:** 1 Department of Thoracic Surgery, University Hospital “St. Ivan Rilski”, Medical University of Sofia, Sofia, BGR; 2 Department of Pulmonary Diseases, University Hospital “St. Ivan Rilski”, Medical University of Sofia, Sofia, BGR; 3 Department of Pathology, University Hospital “St. Ivan Rilski”, Medical University of Sofia, Sofia, BGR

**Keywords:** chest wall resection, localized plasmacytoma, reconstruction, sternum, thoracic surgery

## Abstract

Localized sternal plasmacytoma is a rare and aggressive oncologic condition. Surgical resection followed by radiotherapy offers the highest chance of cure. Radical resection of the chest wall is technically feasible and is associated with improved outcomes. Subsequent reconstruction using meshes, plates, and both autologous and allogenic tissues allows for the restoration of chest wall function and may prolong survival. Patients with solitary plasmacytoma require close follow-up to monitor for local recurrence or distant disease progression. We present a case of a 63-year-old man diagnosed with a differentiated plasmacytoma of the manubrium. Successful resection and anterior chest wall reconstruction were performed.

## Introduction

Sternal tumors are rare entities that present significant diagnostic and therapeutic challenges for both surgeons and oncologists. Solitary plasmacytomas (SP) are rare, accounting for less than 5% of all plasma cell dyscrasias. These neoplasms may be malignant-either primary or secondary-or benign, including entities such as osteochondroma, osteoma, hemangioma, fibrous dysplasia, and Langerhans cell histiocytosis [1,2]. The aim of this report is to describe a rare case of differentiated plasmacytoma involving the manubrium in a 63-year-old man and to discuss its diagnostic and therapeutic implications.

## Case presentation

A 63-year-old man was admitted to the Thoracic Surgery Department of University Multiprofile Hospital for Active Treatment (UMHAT) "Sveti Ivan Rilski" with complaints of a painless sternal mass, first noticed five months earlier, and exertional dyspnea. He reported a significant smoking history (50 pack-years) and chronic alcohol consumption (approximately 100 mL of concentrated alcohol daily). His past medical history included chronic obstructive pulmonary disease (COPD) and chronic ischemic heart disease, for which he was receiving maintenance therapy with tiotropium two inhalations daily, bisoprolol 5 mg once daily, and amlodipine/valsartan/hydrochlorothiazide 160/10/12.5 mg once daily.

**Figure 1:**
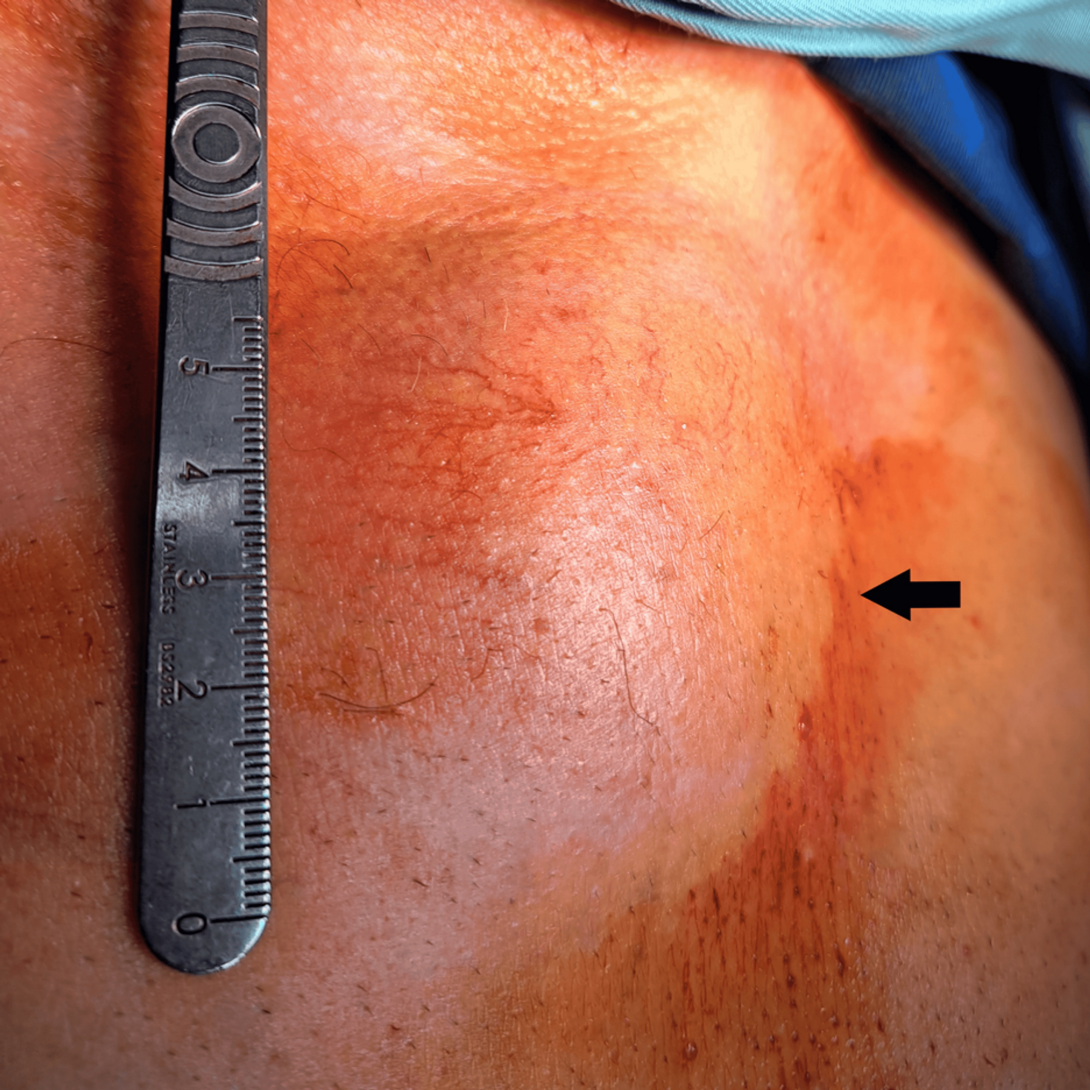
Preoperative photograph showing the manubrial lesion (black arrow indicates the tumor).

On physical examination, a firm, non-tender mass measuring approximately 50 × 50 mm was palpated over the manubrium (Figure 1). Laboratory investigations revealed mild leukocytosis (white blood cell (WBC): 11.18 × 10⁹/L), elevated erythrocyte sedimentation rate (ESR: 40 mm/h), and mild hyperglycemia (7.10 mmol/L) (Table 1). Spirometry and arterial blood gas analysis were within normal limits.

**Table 1:** Laboratory investigations at admission.

Parameters	Patient values	Reference range
White blood cells	11.18	3.50-10.80 x 10^9^/L
Red blood cells	4.22	3.76-5.34 x 10^12^/L
Hemoglobin	140	115-150 g/L
Hematocrit	0.397	0.350-0.490 L/L
Mean corpuscular volume (MCV)	94.0	85.2-98.5 fL
Mean corpuscular hemoglobin (MCH)	33.2	27.0-33.0 pg
Platelets	313	112-330 x 10^9^/L
Lymphocytes (%)	16.4	15.2-41.9
Monocytes (%)	8.9	4.9-11.0
Eosinophils (%)	1.0	Up to 6.2
Basophils (%)	0.1	Up to 1.3
Neutrophils (%)	73.6	37.6-78.7
Lymphocytes, absolute count	1.83	1.00-4.50 x 10^9^/L
Monocytes, absolute count	1.00	0.40-1.10 x 10^9^/L
Eosinophils, absolute count	0.11	0.04-0.50 x 10^9^/L
Basophils, absolute count	0.01	Up to 0.10 x 10^9^/L
Neutrophils, absolute count	8.23	2.00-7.00 x 10^9^/L
Erythrocyte sedimentation rate	40	Up to 30 mm/h
Potassium	4.67	3.5-5.6 mmol/L
Sodium	141.8	135-151 mmol/L
Chloride	111.4	93-112 mmol/L
Glucose	7.10	3.50-6.10 mmol/L
Creatinine	108.80	Up to 134.00 µmol/L
Urea	7.9	1.7-8.2 mmol/L
Aspartate aminotransferase	27	Up to 40.00 U/L
Alanine aminotransferase	17	Up to 41.00 U/L
Total protein	68.8	64-83 g/L

**Figure 2:**
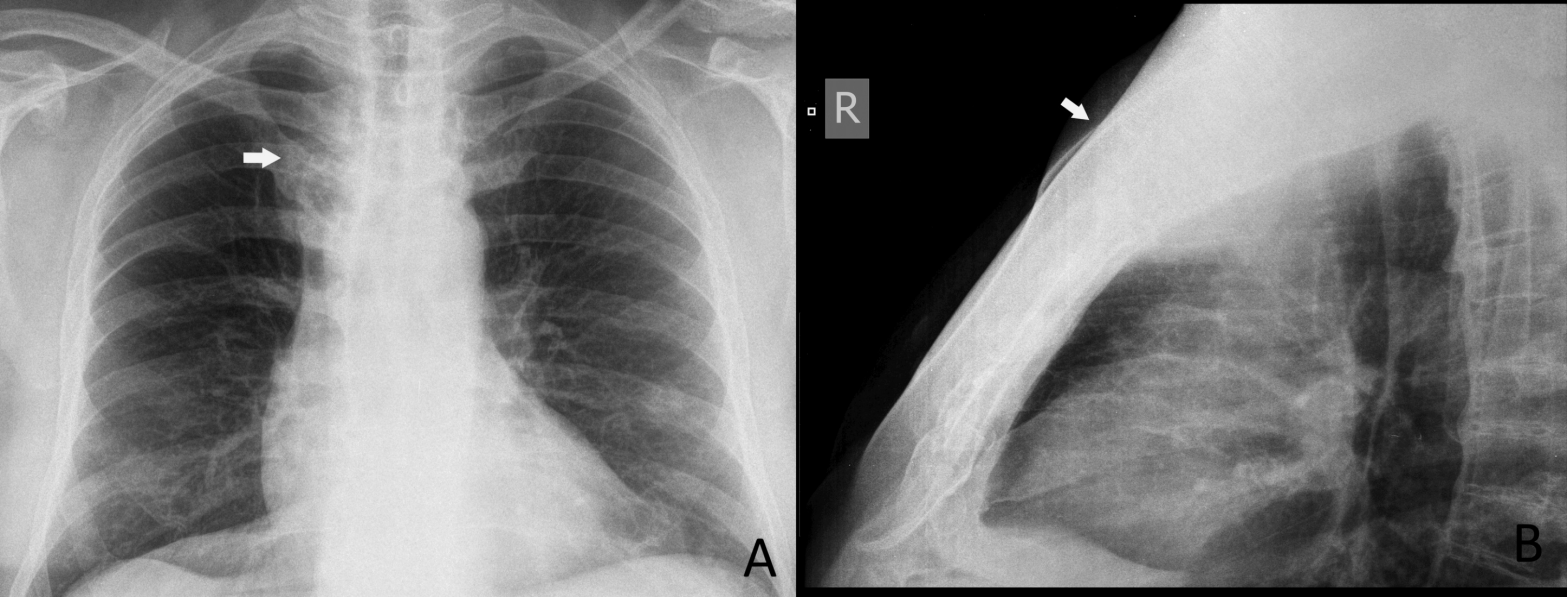
Chest X-rays demonstrating a manubrial opacity (white arrow indicates the tumor). (A) Anteroposterior view; (B) lateral view

**Figure 3:**
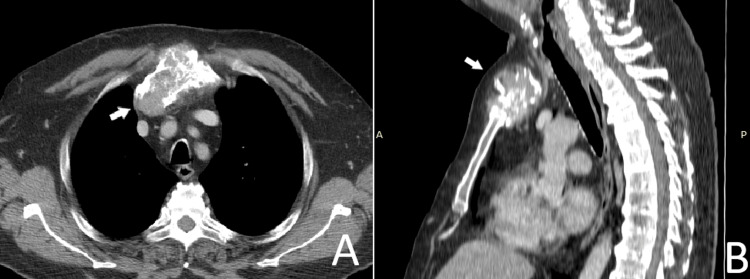
Computed tomography scan showing a manubrial tumor (white arrow indicates the lesion). (A) Axial view; (B) sagittal view

Chest radiography showed an opacity over the manubrium (Figure 2). Computed tomography (CT) revealed an irregularly shaped, infiltrative tumor involving the manubrium, with axial dimensions of 79 × 27 mm (Figure 3). The tumor consisted of both osseous and soft tissue components, some of which protruded into the anterior mediastinum and reached the anterior wall of the innominate vein, without evidence of vascular invasion. The sternal body, clavicles, and adjacent ribs were not affected.

**Figure 4:**
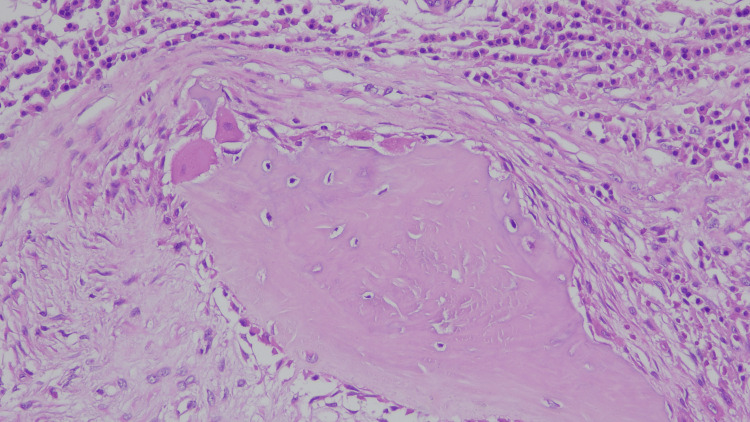
Bone trabecula with osteoblasts lining the surface. Stained with hematoxylin and eosin (H&E), 20×/0.40 magnification

A transthoracic core needle biopsy (tru-cut) of the lesion was performed. Histological evaluation revealed giant osteoblast-like cells, raising suspicion of a malignant mesenchymal neoplasm (Figure 4). The case was reviewed by a multidisciplinary oncological team, and surgical resection was recommended.

**Figure 5:**
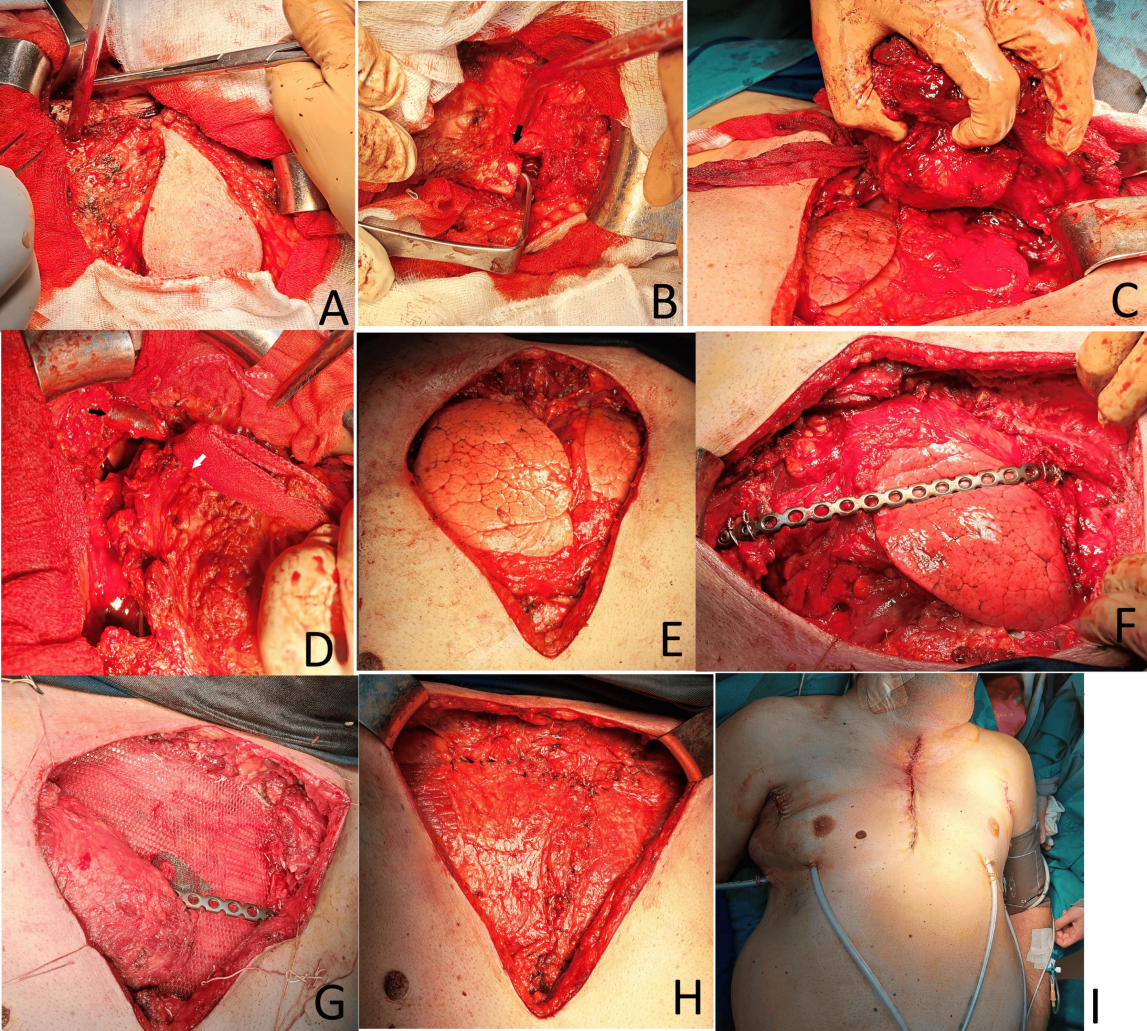
Intraoperative images. (A) Elliptical skin incision over the manubrial tumor; (B) resection of the right clavicle (marked with a black arrow); (C) mobilization of the distal portion of the manubrial lesion; (D) mobilization of the proximal tumor segment (black arrow: right brachiocephalic vein; white arrow: proximal portion of the tumor); (E) resulting postoperative chest wall defect; (F) placement of a titanium plate at the level of the third ribs; (G) placement of a polyester mesh for chest wall reconstruction; (H) coverage of the defect with bilateral myopectoral flaps; (I) final appearance after surgical closure

The procedure began with an elliptical incision around the tumor (Figure 5). The incision was gradually deepened bilaterally and extended inferiorly toward the lower neck. A transverse sternotomy was performed at the level of the fourth intercostal space. The internal mammary arteries and adjacent veins were ligated and transected bilaterally.

The first through fourth ribs were resected bilaterally, along with the corresponding intercostal muscles and the medial portions of the pectoralis major muscles. The medial segments of both clavicles were also excised. The prethyroid muscles and both sternocleidomastoid muscles were disinserted.

**Figure 6:**
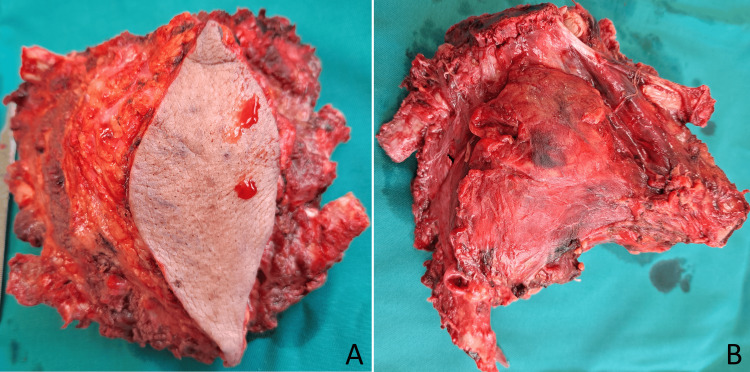
Resected specimen of the manubrial plasmacytoma. (A) Ventral view; (B) dorsal view

The lesion extended into the anterior mediastinum and was closely adherent to the right venous angle and the right brachiocephalic vein. The vein was looped and carefully dissected free using sharp dissection. Radical resection of the tumor was achieved (Figure 6).

The resulting chest wall defect was reconstructed using a polyester mesh, which was secured to the adjacent ribs and the distal sternum. To restore anterior chest wall stability, metal osteosynthesis was performed using a compression plate, four screws, and surgical wires at the level of the third intercostal space.

Muscle flaps were mobilized from the preserved portions of both pectoralis major muscles. Through two additional vertical axillary incisions, the humeral insertions of the muscles were detached. The defect was then covered using the mobilized pectoral muscle flaps.

**Figure 7:**
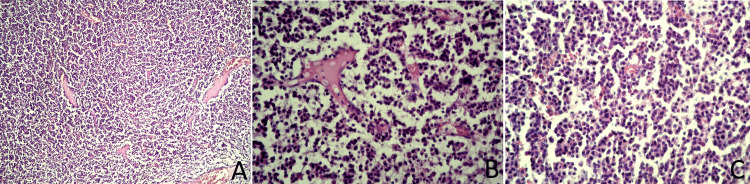
Histopathological images of localized plasmacytoma (hematoxylin and eosin staining). (A) Low magnification (scale bar = 500 µm): diffuse proliferation of monomorphic, plasmacytoid cells associated with osteolysis; (B) medium magnification (scale bar = 200 µm): infiltration of bone tissue by plasmacytoma; a bone trabecula undergoing osteolysis is visible in the center; (C) high magnification (scale bar = 50 µm): diffuse infiltration by tumor cells resembling plasma cells, showing nuclear eccentricity and abundant eosinophilic cytoplasm. Prominent expansion of sinusoidal spaces is also noted

Histopathological examination using hematoxylin and eosin staining revealed a malignant mesenchymal neoplasm of the sternum with infiltrative growth and bone destruction, characterized by the production of osteoid and cartilaginous matrix (Figure 7). Areas of increased cellularity were observed, composed predominantly of rounded, discohesive neoplastic cells with eosinophilic cytoplasm, along with myxoid regions. Numerous multinucleated, osteoclast-like giant cells were seen surrounding structureless osteoid-like material. Tumor infiltration into adjacent muscle bundles was also noted. Surgical resection margins were free of tumor infiltration.

**Figure 8:**
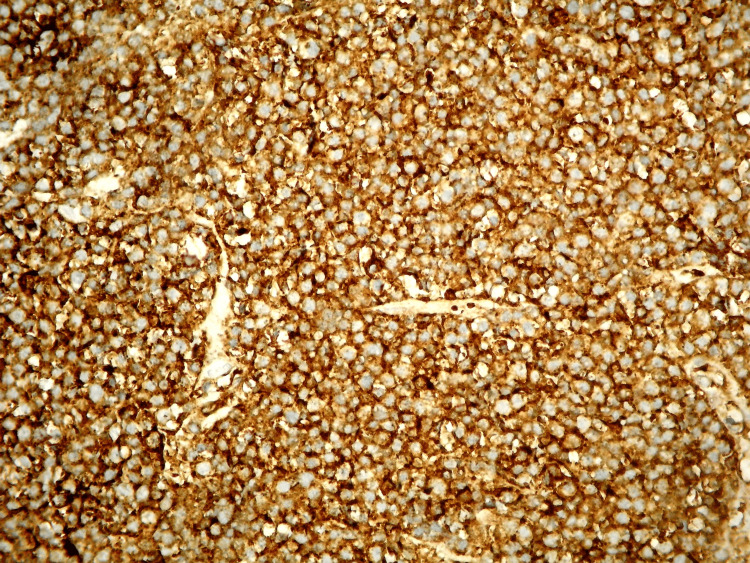
Immunohistochemical staining of sternal plasmacytoma. Strong membranous positivity for CD138 is observed in the tumor cells, confirming their plasmacytic origin

Immunohistochemical analysis demonstrated strong positivity for CD138, with negative staining for CD45 and CD20 (Figure 8). Vangieson staining showed positivity for collagen tissue. Congo red staining, performed to assess for amyloid deposition, was negative. Based on the morphological and immunohistochemical findings, a diagnosis of differentiated localized plasmacytoma of the sternum with diffuse bone involvement was established.

The patient experienced a complicated postoperative course due to significant comorbidities, including COPD and chronic ischemic heart disease, as well as impaired chest wall mechanics, resulting in an ineffective cough and poor expectoration. These factors contributed to a COPD exacerbation and secondary inflammatory complications in the pulmonary parenchyma. As a result, the patient required prolonged mechanical ventilation and underwent tracheostomy on the 25th postoperative day and 11th day after the endotracheal intubation. A 34 Fr tracheostomy tube was placed to facilitate effective airway secretion management.

During the intensive care stay, cardiovascular complications also emerged, including a cardiac arrest due to low-amplitude ventricular tachycardia, which was successfully reversed with two episodes of electrical defibrillation. Additionally, the patient developed paroxysmal supraventricular tachycardia with a heart rate up to 180 bpm, which was pharmacologically controlled with intravenous metoprolol.

Following stabilization of these complications, gradual weaning from mechanical ventilation was initiated. The patient transitioned from invasive mechanical ventilation to non-invasive high-flow oxygen support, and ultimately to spontaneous breathing with effective airway clearance. He was successfully discharged in the third postoperative month (92nd day) and referred to begin adjuvant radiotherapy (RT).

## Discussion

SP are rare, accounting for less than 5% of all plasma cell dyscrasias [2]. They are classified into three subtypes: SP of bone (SPB), extramedullary plasmacytoma, and multiple SP, with SPB representing the majority [2]. SP is considered a localized manifestation of a clonal plasma cell disorder and is regarded as a potential precursor to multiple myeloma (MM), with a median time to progression of approximately five years [1,3]. Progression risk is significantly higher in bone-based lesions compared to extramedullary locations [4]. SPB also carries a worse prognosis than its extramedullary counterpart [4].

Plasmacytomas show a predilection for sites of persistent hematopoietic marrow, such as the sternum [1]. The Surveillance, Epidemiology, and End Results (SEER) database reports a median age at diagnosis of 63 years, a male predominance, and bone involvement in over half of the cases [5]. Favorable prognostic factors include age ≤ 65 years and male sex [6].

Patients with SPB typically present with pain due to bone destruction, mass effect, or pathological fracture [7]. In the present case, the patient reported a painless sternal mass and exertional dyspnea, but delayed seeking medical attention. The diagnosis was further postponed due to a concomitant exacerbation of COPD, which required initial admission to a pulmonology clinic.

Guidelines recommend comprehensive staging of all patients with SP, including bone marrow biopsy, serum studies to exclude systemic involvement, and imaging of the entire skeleton [7]. Advanced imaging, such as whole-body magnetic resonance imaging or positron emission tomography/CT, is advised to rule out multifocal disease [8]. In our case, chest CT and tru-cut biopsy suggested a malignant sternal lesion, raising suspicion of osteosarcoma. Due to the uncertainty of the histology and risk of aggressive behavior, radical resection was performed both for diagnostic clarification and curative intent.

RT remains the most commonly used treatment for SP (48.8%), followed by surgery alone (11.6%) or surgery combined with RT (21.2%) [5]. While European guidelines reserve surgery for cases involving instability, neurological compromise, or impending fracture [8], some studies support combined surgery and RT to optimize local control and survival [4,6]. Radical resection with negative margins (R0) is associated with better outcomes, especially when the tumor involves critical structures such as the heart, major vessels, or airways [9]. Sternectomy, when performed with curative intent, yields five- and 10-year overall survival rates of 67% and 58%, respectively [10].

Reconstruction following chest wall resection aims to restore skeletal stability, prevent paradoxical chest movement, maintain respiratory mechanics, and ensure adequate cosmesis [11]. Use of prosthetic meshes, titanium plates, and muscle flaps, as in the present case, allows for functional recovery and improved quality of life.

RT with doses of ≥40 Gy in 20 fractions and a margin of at least 2 cm is recommended for SPB, achieving local control rates above 80% [7]. Combined modality treatment (surgery and RT) appears particularly beneficial in younger male patients [5].

The role of chemotherapy in SP remains unclear. It may be considered in high-risk patients with residual disease after RT or rapid progression [12]. Overall, the five-year survival for SPB exceeds 90%, and median progression-free survival following diagnosis is 61 months [3].

## Conclusions

Localized sternal plasmacytoma is a rare and potentially aggressive malignancy that has been discussed. Surgical resection followed by RT offers the best chance for cure and long-term disease control. Radical chest wall resection is technically feasible and can lead to favorable outcomes when combined with appropriate reconstruction using meshes, plates, and autologous or allogenic tissues to restore structural integrity and respiratory function. Close long-term follow-up is essential to monitor for local recurrence or progression to systemic disease.
